# Parrots do not show inequity aversion

**DOI:** 10.1038/s41598-019-52780-8

**Published:** 2019-11-11

**Authors:** Anastasia Krasheninnikova, Désirée Brucks, Nina Buffenoir, Dániel Rivas Blanco, Delphine Soulet, Auguste von Bayern

**Affiliations:** 1 Max-Planck-Institute for Ornithology, Eberhard-Gwinner-Str., 82319 Seewiesen, Germany; 2 Max-Planck Comparative Cognition Research Station, Loro Parque Fundacíon, 38400 Puerto de la Cruz Tenerife, Spain; 3 Department of Biology, Ludwig-Maximilians-University of Munich, 82152 Planegg-Martinsried, Germany

**Keywords:** Animal behaviour, Behavioural ecology

## Abstract

Inequity aversion, the negative reaction to unequal treatment, is considered a mechanism for stabilizing cooperative interactions between non-kin group members. However, this might only be adaptive for species that switch cooperative partners. Utilizing a comparative approach, inequity aversion has been assessed in many mammalian species and recently also in corvids and one parrot species, kea, revealing mixed results. To broaden our knowledge about the phylogenetic distribution of inequity aversion, we tested four parrot species in the token exchange paradigm. We varied the quality of rewards delivered to dyads of birds, as well as the effort required to obtain a reward. Blue-headed macaws and African grey parrots showed no reaction to being rewarded unequally. The bigger macaws were less willing to exchange tokens in the “unequal” condition compared to the “equal high” condition in which both birds obtained high quality rewards, but a closer examination of the results and the findings from the control conditions reveal that inequity aversion does not account for it. None of the species responded to inequity in terms of effort. Parrots may not exhibit inequity aversion due to interdependence on their life-long partner and the high costs associated with finding a new partner.

## Introduction

Receiving equal outcomes for equal cooperative effort is a basic expectation in our everyday lives. If this premise is violated by rewarding individuals unequally for the equal amount of work, a negative behavioural reaction, termed inequity aversion, arises^1^. This aspiration for equity has been proposed to be a driving force in the maintenance of cooperative relationships not only in humans but also in non-human animals (see^2^ for a review), since unequal cooperative interactions are not adaptive in the longer term. For many species, however, it remains unknown whether their natural cooperative behaviour is sufficiently diverse, i.e., whether they engage in cooperative interactions with a variety of different partners in various contexts, so as to render a general aversion to inequity beneficial.

Traditionally, inequity aversion has been assessed in an experiment setup, in which two (trained) individuals, placed in adjacent compartments, hand back tokens to an experimenter alternatingly in exchange for a reward. The test subjects can therefore observe whether their partner is rewarded equally for handing back the tokens or not. The inequity is induced either by handing out rewards of different quality (low quality vs. high quality reward), quantity (rewarded vs. not rewarded), or by requesting different effort from both individuals before they receive their reward (e.g. asking for multiple exchanges vs. rewarding without any exchange). Inequity aversion is thought to show when individuals refuse to exchange tokens or accept rewards, upon being rewarded unequally or having invested unequal effort. Note however that there are also other methodologies to evaluate inequity aversion (see ref.^3^ for a review).

Using the token exchange paradigm various primate species have been tested in the last decade, revealing some variation in their response to inequity. While for example, capuchin monkeys (*Cebus spec*.)^4^, chimpanzees (*Pan troglodytes*)^5^, rhesus macaques (*Macaca mulatta*)^6^, and potentially bonobos (*Pan paniscus*)^7^, exhibited inequity aversion when rewarded unequally for performing the same action, orang-utans (*Pongo pygmaeus*)^8^, marmosets (*Callithrix jacchus*), squirrel monkeys (*Saimiri boliviensis*) and owl monkeys (*Aotus spp*.)^9^ did not show an aversion to being rewarded unequally. Note, however, that it remains controversial whether non-human primates truly show inequity aversion. Some of the previous evidence has been called into question due to conflicting results yielded by subsequent studies. For example, employing a different methodology, Bräuer and colleagues^7^ and Silberberg and colleagues^10^ failed to replicate the evidence for inequity aversion reported by earlier studies in chimpanzees and capuchins, respectively. These contrasting findings suggest that nonhuman inequity aversion may be less robust than indicated by initial findings (ref. ^11^) and led to an extensive debate over whether the exchange task really measures inequity aversion, or merely frustration about not receiving a reward (see for example ref. ^12^). It should also be noted that, recently, the “Social Disappointment Hypothesis” has been put forward providing an alternative explanation for the responses of primates in food donation tasks^13^. Engelmann and colleagues^13^ found that chimpanzees refused to participate in a task significantly more often if a human experimenter was handing out rewards than if the rewards were delivered by an automatic food dispenser without an experimenter present. Moreover, the subjects were more likely to reject food in the presence of the human experimenter when the conspecific partner was absent than if it was present, suggesting that their reactions to unequal treatment were explained by disappointment in the human experimenter for not treating them with better rewards, rather than being disappointed in how they are being treated in comparison to another individual, as the inequity hypothesis predicts.

Nevertheless, it has been proposed that species that rely on cooperative interactions with varying non-kin group members may benefit from evaluating the equality of their cooperative payoffs in order to assess whether to stick with a certain partner or to look for another one with whom to gain better outcomes^14^. This hypothesis has gained further support by studies on non-primate species; dogs^15^, wolves, *Canis lupus*^16^ and rats, *Rattus norvegicus*^17^, all gregarious species that regularly cooperate with non-kin group members, have shown inequity aversion in paradigms adapted to their species-specific abilities. In contrast, species which are mostly reliant on a single cooperative partner, such as long-term monogamous species that rely on bi-parental care^9,18^, might not show sensitivity to unequal payoffs, because they exhibit such high levels of mutual interdependence that looking for a better partner would not pay off^9,14^. Likewise, on the other end of the social spectrum, less gregarious species do not require such a mechanism since assessments of cooperative payoffs are not relevant in their everyday life^8^.

In order to examine this hypothesis further and to gain deeper insights into the phylogenetic distribution of inequity aversion, it is revealing to broaden the comparison to species outside of the mammalian order. Recently, a few first studies have tested inequity aversion in birds, namely in a few corvid species and one parrot species. Corvids and parrots are particularly interesting models, because they stand out among birds and more generally among vertebrates, for their enlarged and densely packed brains, complex cognition and social complexity, hence exhibiting many similarities to primates^19–21^. In contrast to most primates however, the majority of both corvid and parrot species exhibits long-term monogamy^22^. For example, in the typical exchange task, ravens (*Corvus corax*) and carrion crows (*Corvus corone*) reacted to an unequal reward distribution refusing to exchange if they received a reward of lower quality than their partner (note that the study did not rule out completely that this reaction was governed by frustration however). Additionally, the corvids stopped participating if their partner received a reward for ‘free’ while they had to perform the exchange action as before, hence displaying sensitivity to unequal effort^23^. On the contrary, New Caledonian crows (*Corvus moneduloides*) did not respond to unequal treatment^24^, albeit in a very different cooperative paradigm, thus the difference in methodology makes the findings across these corvids less comparable. In parrots, so far only kea (*Nestor notabilis*) from the superfamily *Strigopoidea* have been tested in a classic token exchange paradigm, and their reactions to inequity in terms of quality (food quality and absence of reward) and effort were assessed^25^. The kea continued to exchange tokens irrespective of whether their partner received a reward of higher value or was rewarded for free (whilst they had to exchange a token), thus showing no inequity aversion.

The objective of the current study was to provide further data on the phylogenetic distribution of inequity aversion in species distantly related to primates. At the same time it aimed at providing more data on inequity aversion in long-term monogamous species, by testing the reaction to inequity of four parrot species belonging to the *Psitta*c*oidea* superfamily, namely great green macaws (*Ara ambiguus)*, blue-throated macaws (*Ara glaucogularis)*, blue-headed macaws (*Primolius couloni*) and African grey parrots (*Psittacus erithacus*). All of these species form long-term monogamous pairs and live in family groups, while exhibiting fission-fusion dynamics^26–29^. Both parents provide food to their young and the offspring stays with their parents for at least one breeding season. The degree of sociality might differ between the four species, as African grey parrots have been observed in much larger flocks^28^ than *P. couloni*^27^, *A. glaucogularis*^29^ and *A. ambiguus*^26^, although group size is not necessarily a reliable indicator of social complexity^20^. Besides, the recently reported flock sizes might not be representative, particularly for the two *Ara* species, considering that they are threatened by extinction and only small numbers of individuals remain in the wild.

The parrots were tested using a token-exchange paradigm^4^, in which two individuals were requested alternately to hand back tokens in exchange for a food reward. Depending on the test condition, they were rewarded for performing this action either equally or unequally. With difference to previous studies, the equal or unequal rewards were only handed out simultaneously, once both subjects had given their token to the experimenter, rather than rewarding them alternatingly for their respective exchange, in order to increase the salience of the social comparison. Furthermore, we incorporated control conditions to disentangle effects of frustration and motivation from a reaction to inequity (see Tables 1 and 2). Additional conditions tested whether varying the effort required for receiving a reward elicited an inequity response. Considering the first results of kea and the fact that parrots form valuable long-term pair-bonds and probably cooperate primarily with their mate, we hypothesize that parrots do not exhibit inequity aversion.

**Table 1:** Overview of and rationale for the test conditions.

	Subject	Partner	Effort	Rationale
***Reward variation***				
Equal High (EQUH)	HQR	HQR	*Subject* and *partner* exchange token once	Baseline motivation to *work for HQR when payoff is equal*.
Equal Low (EQUL)	LQR	LQR		Baseline motivation to *work for LQR when payoff is equal*.
Unequal (UNEQ)	LQR	HQR		Test for inequity aversion; is the motivation to work for LQR lowered when *the partner receives a better payoff* compared to the baselines (EQUH & EQUL)?
Food Control (FC)	LQR	HQR* (partner absent)		Serves as comparison to UNEQ where the partner receives a HQR (better payoff), whereas, here, the HQR is delivered to the *empty* neighbour compartment. Is the subject’s motivation lowered just by a visible better reward, compared to when a partner receives it?
***Effort variation***				
Effort Control (EC)	LQR	— (partner absent)	*Subject*: 2x exchange for one reward *Partner:* reward for free	Baseline motivation to perform *multiple exchanges*.
Unequal Effort (UNEF)	LQR	LQR		Test for inequity aversion; is the motivation lowered when the partner needs to invest *less effort for same payoff?*

*Reward is placed in the partner’s compartment and removed again after 5 s.

**Table 2:** Summary of the predictions for the three main variables in the experimental conditions according to the hypothetical framework of inequity aversion.

Variable	Prediction	Prediction	Prediction
	Inequity tolerant	Inequity averse	Frustration
*Exchanges*	**EQUH** = **UNEQ**	**EQUH** > **UNEQ**	EQUH > UNEQ
	EQUL ≥ UNEQ	EQUL > UNEQ	EQUL > UNEQ
	FC = UNEQ	FC > UNEQ	**FC** = **UNEQ**
*Latency to exchange/ Latency to accept reward*	**EQUH** = **UNEQ**	**EQUH** < **UNEQ**	EQUH < UNEQ
	EQUL ≤ UNEQ	EQUL ≤ UNEQ	EQUL < UNEQ
	FC = UNEQ	FC < UNEQ	**FC** = **UNEQ**

Most important distinctions between predictions are indicated in bold.

## Results

**Figure 1:**
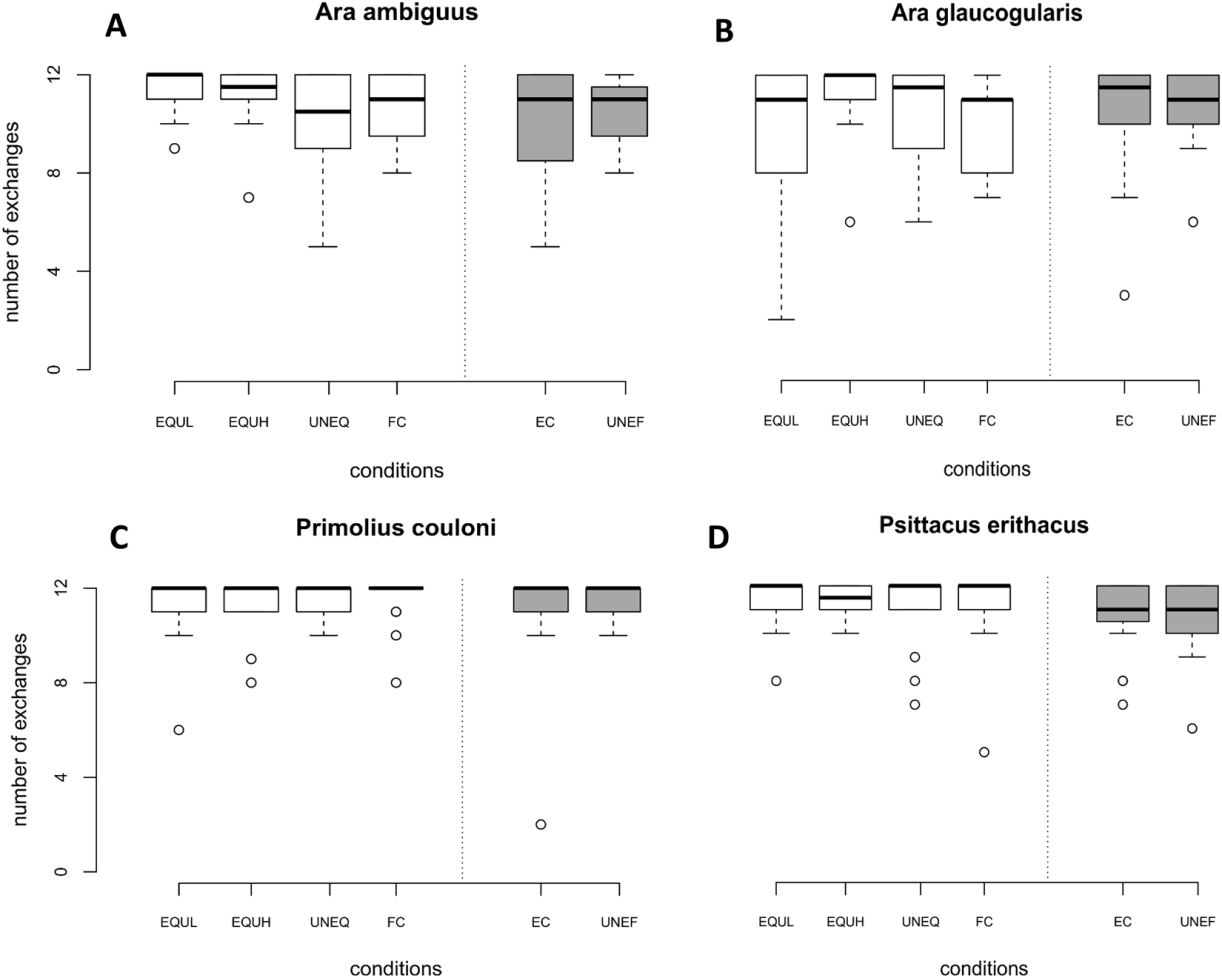
(**A**–**D**) Exchanges across test conditions separately for each species (all test sessions combined; EQUL = equal low, EQUH = equal high, UNEQ = unequal, FC = food control, EC = effort control, UNEF = unequal effort).

We found a species-condition interaction on the number of exchanges (Wald χ^2^ = 64.06, df = 15, p < 0.001) and analysed the species separately (see Fig. 1a–d). For a summary of significant differences for specified variables between conditions for each parrot species see Table 3.

**Table 3:** Summary of significant differences for specified variables between conditions for each parrot species.

Variable	Species			
	*A. ambiguus*	*A. glaucogularis*	*P. couloni*	*P. erithacus*
Exchanges	EQUH > UNEQ	EQUH > UNEQ*	EC < UNEF*	
	EQUL > UNEQ	EQUL < UNEQ*		
Latency to exchange	EQUH < UNEQ	EQUH < UNEQ	EQUH < UNEQ	EQUH < UNEQ
	EQUL < UNEQ			FC > UNEQ
Latency to accept reward	EQUH < UNEQ	EQUH < UNEQ	EQUH < UNEQ	FC > UNEQ
	EQUL < UNEQ	EQUL < UNEQ	FC > UNEQ	
	FC > UNEQ			

*Only in the third test session.

### Great green macaws *(Ara ambiguus)*

The great green macaws did not show any session effect on the number of exchanges performed across test sessions (Wald χ^2^ = 0.08, *df* = 1, *p* = 0.781); however, the number of exchanges differed between test conditions (Wald χ^2^ = 32.76, *df* = 5, *p* < 0.001). In particular, the parrots performed more exchanges in the equal low (EQUL) and equal high (EQUH) conditions (i.e. when both parrots received the same type of reward of either high or low value) than in the unequal condition (UNEQ; one parrot was rewarded with low value food while the other received a high value reward) (GLMMs: EQUL: *β* = 1.32 ± 0.32, *z* = 4.14, *p* < 0.001; EQUH: *β* = 0.94 ± 0.28, *z* = 3.32, *p* < 0.001; see Fig. 1a). The number of exchanges did not differ between the unequal condition and the food control (FC) where no other bird was present (GLMM: *β* = 0.39 ± 0.25, *z* = 1.57, *p* = 0.116). Furthermore, the great green macaws did not react to differences in effort, given that the number of exchanges did not differ between the unequal effort (UNEF; one parrot has to exchange twice while the partner gets a low value reward for free) and the effort control (EC; partner absent, the parrot needs to exchange twice for a low value reward) (GLMM: *β* = −0.32 ± 0.23, *z* = −1.38, *p* = 0.169).

Likewise, the latency to exchange tokens (log-transformed) differed between conditions (Anova: *F* = 10.41, *df* = 5, *p* < 0.001) but was not affected by session number (Anova: *F* = 0.92, *df* = 1, *p* = 0.338). In particular, in the unequal condition (mean ± SD: 4.23 ± 2.39 s) the parrots took longer to exchange tokens in comparison to the EQUL (2.21 ± 1.65 s; LM: *β* = −0.71 ± 0.18, *t* = −4.05, *p* < 0.001) and EQUH condition (1.41 ± 0.81 s; LM: *β* = −1.11 ± 0.18, *t* = −6.33, *p* < 0.001). No differences in the latencies to exchange were observed between the unequal and FC condition (3.86 ± 2.26 s; LM: *β* = −0.14 ± 0.18, *t* = −0.77, *p* = 0.442) and between the unequal effort (2.65 ± 1.59 s) and EC condition (2.73 ± 1.71 s; LM: *β* = −0.03 ± 0.18, *t* = −0.17, *p* = 0.867).

It rarely happened that the great green macaws refused rewards (mean ± SD: 0.06 ± 0.27 refusals) and accordingly, we were not able to analyse this variable. Nonetheless, the latency to accept rewards (1/square root transformed) differed between conditions (Anova: *F* = 12.91, *df* = 5, *p* < 0.001) but not test sessions (Anova: *F* = 0.35, *df* = 1, *p* = 0.556). The parrots accepted rewards slower in the unequal condition (mean ± SD: 1.30 ± 1.26 s) compared to the EQUL (0.57 ± 0.17 s; LM: *β* = 0.26 ± 0.08, *t* = 3.15, *p* = 0.002) and EQUH condition (0.51 ± 0.22 s; LM: *β* = 0.38 ± 0.08, *t* = 4.59, *p* < 0.001). Furthermore, the parrots accepted rewards faster in the unequal condition compared to the non-social FC condition (2.04 ± 1.63 s; LM: *β* = −0.21 ± 0.08, *t* = −2.54, *p* = 0.012). No differences emerged between the unequal effort condition (0.62 ± 0.23 s) and EC (0.82 ± 0.58 s; LM: *β* = −0.13 ± 0.08, *t* = −1.56, *p* = 0.122).

### Blue-throated macaws *(Ara glaucogularis)*

For the blue-throated macaws, the number of exchanges differed across test sessions, while some conditions were affected more strongly than others (session - condition interaction: Wald χ^2^ = 20.00, *df* = 5, *p* = 0.001). This effect was mainly driven by their behaviour in the EQUL and EC conditions, in which the parrots decreased their exchanges across test sessions (GLMMs: EQUL: *β* = −0.71 ± 0.35, *z* = −2.02, *p* = 0.043; EC: *β* = −1.04 ± 0.38, *z* = −2.76, *p* = 0.006). Analysing each session separately, we found no differences between conditions for the first and second session (1^st^ session: Wald χ^2^ = 10.64, *df* = 5, *p* = 0.059; 2^nd^ session: Wald χ^2^ = 3.99, *df* = 5, *p* = 0.551), but only for the third session (Wald χ^2^ = 25.19, *df* = 5, *p* < 0.001). Specifically, the parrots performed more exchanges in the EQUH condition compared to the unequal condition (GLMM: *β* = 2.49 ± 1.06, *z* = 2.35, *p* = 0.019), while they performed fewer exchanges in the EQUL condition compared to the unequal condition (GLMM: *β* = −1.28 ± 0.44, *z* = −2.95, *p* = 0.003; see Fig. 1b). The number of exchanges did not differ between the FC and unequal condition (GLMM: *β* = −0.12 ± 0.48, *z* = −0.24, *p* = 0.807) and between the EC and unequal effort condition (GLMM: *β* = −0.90 ± 0.48, *z* = −1.87, *p* = 0.061).

Furthermore, when analysing the latencies to exchange the tokens (1/square-root transformed), we found that the latencies did not differ across test sessions (Anova: *F* = 1.60, *df* = 1, *p* = 0.209), but only between conditions (Anova: *F* = 6.27, *df* = 5, *p* < 0.001). The blue-throated macaws exchanged tokens slower in the unequal condition (mean ± SD: 2.69 ± 2.69 s) compared to the EQUH condition (1.00 ± 0.24 s; LM: *β* = 0.24 ± 0.07, *t* = 3.37, *p* = 0.001). However, we found no differences in the latencies to exchange in the EQUL (3.56 ± 3.61 s) and FC condition (3.50 ± 3.47 s) compared to the unequal condition (LMs: EQUL–UNEQ: *β* = −0.07 ± 0.07, *t* = −0.95, *p* = 0.346; FC–UNEQ: *β* = −0.10 ± 0.07, *t* = −1.42, *p* = 0.158). Likewise, the latency to exchange did not differ between the unequal effort condition (2.22 ± 0.85 s) and EC (1.81 ± 0.89 s; LM: *β* = 0.11 ± 0.07, *t* = 1.49, *p* = 0.138).

The blue-throated macaws rarely refused rewards (mean ± SD: 0.02 ± 0.14 refusals), consequently we were not able to analyse this variable. The latency to accept the reward (1/square-root transformed) did differ across conditions (Anova: *F* = 8.18, *df* = 5, *p* < 0.001), but not across test sessions (Anova: *F* = 0.99, *df* = 1, *p* = 0.322). The birds accepted the rewards slower in the unequal condition (1.30 ± 0.72 s) compared to the EQUH (0.74 ± 0.11 s; LM: *β* = 0.21 ± 0.06, *t* = 3.55, *p* < 0.001) and EQUL condition (0.86 ± 0.25 s; LM: *β* = 0.16 ± 0.06, *t* = 2.58, *p* = 0.011). No differences in the latency to accept rewards were observed between the unequal and FC condition (2.31 ± 2.37 s; LM: *β* = −0.09 ± 0.06, *t* = −1.46, *p* = 0.148) and between the unequal effort (0.81 ± 0.17 s) and EC condition (0.80 ± 0.89 s; LM: *β* = 0.01 ± 0.06, *t* = 0.15, *p* = 0.879).

### Blue-headed macaws (*Primolius couloni)*

We found a session – condition interaction in the blue-headed macaws (Wald χ^2^ = 13.02, *df* = 5, *p* = 0.023) and subsequently analysed each session separately. The results revealed that the number of exchanges did not differ across conditions in the first and second test session (1^st^ session: Wald χ^2^ = 7.47, *df* = 5, *p* = 0.188; 2^nd^ test session: Wald χ^2^ = 10.54, *df* = 5, *p* = 0.061). In the third test session, however, we found an effect of condition on the number of exchanges (Wald χ^2^ = 22.01, *df* = 5, *p* < 0.001). The parrots did not react to inequity and performed equal numbers of exchanges in the equal conditions and FC condition compared to the unequal condition (GLMMs: EQUL: *β* = −0.72 ± 1.27, *z* = −0.56, *p* = 0.573; EQUH: *β* = 0.01 ± 1.46, *z* = 0.01, *p* = 0.996; FC: *β* = −0.72 ± 1.27, *z* = −0.56, *p* = 0.573; see Fig. 1c). Interestingly, the condition effect was mainly driven by the conditions involving effort, in which fewer exchanges were performed in the EC compared to the unequal effort condition (GLMM: *β* = −2.80 ± 0.00, *z* = −785.7, *p* < 0.001).

The blue-headed macaws exhibited differential latencies to exchange the tokens (1/square-root transformed) across conditions (Anova: *F* = 7.90, *df* = 5, *p* < 0.001), but not across test sessions (Anova: *F* = 0.00, *df* = 1, *p* = 0.958). In particular, they exchanged slower during the unequal (mean ± SD: 2.29 ± 1.47 s) compared to the EQUH condition (1.22 ± 0.46 s; LM: *β* = 0.20 ± 0.06, *t* = 3.65, *p* < 0.001), however, the latencies to exchange did not differ between the EQUL (2.14 ± 1.33 s) and FC condition (2.81 ± 1.87 s) compared to the unequal condition (LMs: EQUL-UNEQ: *β* = 0.01 ± 0.06, *t* = 0.13, *p* = 0.900, FC-UNEQ: *β* = −0.07 ± 0.06, *t* = −1.30, *p* = 0.195). Likewise, we detected no differences in the latency to exchange between the unequal effort (2.15 ± 0.92 s) and EC condition (2.90 ± 1.21 s; LM: *β* = −0.10 ± 0.06, *t* = −1.71, *p* = 0.090).

Like the other parrot species, the blue-headed macaws only rarely refused rewards (mean ± SD: 0.07 ± 0.28 refusals), thus not allowing for an analysis of this variable. Nonetheless, we found differences in the latency to accept the reward (log-transformed) across test conditions (Anova: *F* = 5.98, *df* = 5, *p* < 0.001), but not across test sessions (Anova: *F* = 0.89, *df* = 1, *p* = 0.349). The latency to accept rewards was longer in the unequal (1.76 ± 1.07 s) compared to the EQUH condition (0.93 ± 0.20 s; LM: *β* = −0.50 ± 0.16, *t* = −3.24, *p* = 0.002). No differences emerged between the unequal and EQUL condition (1.64 ± 0.72 s; LM: *β* = 0.00 ± 0.16, *t* = −0.01, *p* = 0.990), while the birds accepted the rewards faster in the unequal compared to the FC condition (2.52 ± 1.55 s; LM: *β* = 0.34 ± 0.16, *t* = 2.17, *p* = 0.032). The birds accepted rewards equally quickly in the unequal effort (1.63 ± 0.73 s) and the EC condition (1.51 ± 0.58 s; LM: *β* = −0.05 ± 0.16, *t* = −0.31, *p* = 0.757).

### African grey parrots (*Psittacus erithacus)*

The African greys’ exchange behaviour was affected by the session number (Wald χ^2^ = 9.12, *df* = 1, *p* = 0.003), as well as by the test condition (Wald χ^2^ = 29.54, *df* = 5, *p* < 0.001). The grey parrots increased the number of their exchanges across test sessions (GLMM: *β* = 0.35 ± 0.11, *z* = 3.02, *p* = 0.003). They were not affected by inequity in the reward distribution and did not decrease exchanges in the unequal condition compared to the equal conditions (GLMMs: EQUL: *β* = 0.64 ± 0.39, *z* = 1.66, *p* = 0.097; EQUH *β* = 0.31 ± 0.35, *z* = 0.88, *p* = 0.379; see Fig. 1d). Moreover, they did not exchange more frequently in the FC condition compared to the unequal condition (GLMM: *β* = 0.24 ± 0.35, *z* = 0.70, *p* = 0.487). Finally, the grey parrots did not react to inequity in terms of effort, as they exchanged tokens equally often in the unequal effort and EC condition (GLMM: *β* = 0.36 ± 0.26, *z* = 1.41, *p* = 0.158).

The African grey parrots exhibited differences in the latency to exchange tokens (log-transformed) across test conditions (Anova: *F* = 7.78, *df* = 5, *p* < 0.001), but the latency did not change across test sessions (Anova: *F* = 0.00, *df* = 1, *p* = 0.993). The parrots exchanged tokens slower during the unequal (mean ± SD: 1.68 ± 0.93 s) compared to the EQUH condition (1.05 ± 0.51 s; LM: *β* = −0.47 ± 0.15, *t* = −3.05, *p* = 0.003), however, we detected no differences between the unequal and EQUL condition (1.60 ± 0.97 s; LM: *β* = −0.08 ± 0.15, *t* = −0.50, *p* = 0.617). The latency to hand back tokens was longer in the FC condition (2.43 ± 1.56 s) than in the unequal condition (LM: *β* = 0.30 ± 0.15, *t* = 1.98, *p* = 0.049). We detected no differences between the latencies to exchange in the unequal effort (1.91 ± 0.81 s) and the EC condition (2.45 ± 1.41 s; LMM: *β* = 0.20 ± 0.15, *t* = 1.28, *p* = 0.202).

In comparison to the other species, the African greys almost never refused to accept a reward (mean ± SD: 0.01 ± 0.08 refusals). However, their latency to accept rewards was affected by the test condition (Anova: *F* = 4.00, *df* = 5, *p* = 0.002) but not by the test session (Anova: *F* = 3.01, *df* = 2, *p* = 0.085). The grey parrots accepted rewards faster in the unequal (0.39 ± 0.30 s) compared to the FC condition (0.63 ± 0.80 s; GLS: *β* = 0.24 ± 0.11, *t* = 2.18, *p* = 0.031), while they exhibited no differences in the latency to accept rewards in the EQUH (0.22 ± 0.14 s) and EQUL (0.25 ± 0.17 s) compared to the unequal condition (GLSs: EQUH-UNEQ: *β* = −0.17 ± 0.11, *t* = −1.49, *p* = 0.139; EQUL-UNEQ: *β* = −0.14 ± 0.11, *t* = −1.29, *p* = 0.200). Likewise, the latency to accept rewards was the same in the two conditions involving effort (EC: 0.23 ± 0.13 s; unequal effort: 0.32 ± 0.29 s; GLS: *β* = −0.09 ± 0.11, *t* = −0.83, *p* = 0.408).

## Discussion

The current study assessed responses to unequal treatment in four parrot species utilizing the classic token-exchange paradigm. We found that the blue-headed macaws and the African grey parrots exhibited no reaction to unequal treatment and continued to exchange tokens independent of the reward distribution. The bigger macaws were less willing to exchange tokens in the “unequal condition”, where their partners received rewards of higher quality than they did, compared to an “equal high” condition in which both birds obtained high quality rewards. However, inequity aversion did not seem to account for the response of either species.

Reactions to unequal treatment are thought to be based on a social comparison between one’s own outcome and that of one’s partner; nonetheless, different underlying mechanisms can give rise to seemingly similar behavioural patterns^30^. As indicated above, the two bigger macaw species refused to exchange tokens and also exhibited a longer latency to exchange tokens and accept rewards in the unequal condition compared to the equal high condition. However, only the great green macaws exhibited this response irrespective of reward quality in the “equal condition”, i.e. also when it was “equally low”. Yet, at a closer look, the food control revealed that the great green macaws were most likely responding to the presence of the high-quality reward rather than to the partner receiving it. They refused to exchange irrespective of whether the high-quality reward was placed into the empty partner’s compartment (food control condition) or was consumed by the partner (unequal condition).

The blue-throated macaws per contra showed the opposite pattern, namely they exchanged less often in the equal low condition compared to the unequal condition, which indicates that their response was not motivated by comparing the outcomes. In contrast to the great green macaws, which remained motivated to exchange tokens also for the low-quality reward over a longer period of time, the blue-throated macaws’ motivation decreased across test sessions, in particular in the equal low and effort control condition, suggesting that they got increasingly frustrated to work for low-quality rewards. Puzzlingly though, this pattern did not prevail in the unequal condition, where they also received only low-quality rewards, but where their partner received high-quality rewards. Here, the blue-throated macaws’ motivation to exchange did not decrease across sessions. An explanation may be that simply seeing the partner receive a high-quality reward in the unequal condition might have sufficed to offset their frustration of working for low-quality rewards and to make them participate again, potentially due to food competition or just seeing the high-quality reward moved.

Opposite to what one would predict if the parrots acted genuinely inequity averse, the great green macaws furthermore took longer to accept the reward in the non-social food control condition compared to the unequal condition. This indicates that some social factor, such as food competition and/or social facilitation might play an additional role, which may potentially even confound some degree of inequity aversion in a species. For example, one might speculate that the parrots may have consumed their reward quickly during the unequal condition due to fear of their partner stealing it or rather due to seeing their partner eating in the first place, while only in the non-social food control condition, the parrots were actually attending to the reward distribution. Competition over food and social facilitation of eating have been reported to play a role in the expression of inequity aversion previously, e.g., capuchin monkeys consumed food faster in the presence of a conspecific who was eating a better reward, compared to the presence of a conspecific who could not access food^31,32^. Interestingly, the same pattern was found in grey parrots and blue-headed macaws, which did not show any signs of inequity aversion, likely reflecting social facilitation too. However, although less parsimonious, an alternative interpretation one may raise is that parrots might be so prosocial that they respond less aversely if a high-quality reward is given to their partner than if the food is just passively placed into the chamber without being fed to any bird.

Interestingly, all of the parrot species seemed to be sensitive to differences in reward quality, hence confirming that they witnessed that their partner had received a reward of different quality in the unequal condition (which had not been warranted by some previous studies using the same paradigm, thus confounding the results, e.g.^10,23^). In particular in the non-social food control condition, the two bigger macaw species refused to exchange tokens if a better reward was delivered to the empty partner’s enclosure, while the smaller parrot species did not refuse to exchange tokens but took longer to do so (African grey parrots) or to accept rewards (blue-headed macaws). Interestingly, while the blue-throated macaws got more frustrated with the test procedure (see discussion above), the African grey parrots showed the opposite pattern and increased their number of exchanges in all conditions across the course of the study. This might indicate that they got used to the exchange procedure and therefore stopped caring about the rewards and their distribution. Accordingly, the sensitivity to reward quality and frustration/motivation levels potentially differ strongly between parrot species, which calls for a careful interpretation of the results from comparative studies.

In contrast to previous findings in capuchin monkeys^4^, where the failure to complete exchanges was roughly equally divided between no token exchange and no food acceptance, it rarely happened that our birds refused to accept rewards. In order to inhibit a previously reinforced behaviour (i.e. an exchange) subjects require inhibitory skills and in order to refuse a reward, they must be able to forgo a directly accessible food, which is a key aspect of delay of gratification^33^. Inhibitory control has been suggested to be an important cognitive prerequisite underlying response to inequity^34^. Indeed, a recent study found that inhibition abilities accounted for individual variation in the reaction to unequal treatment of dogs^35^. Dogs that showed better inhibitory control abilities in a test battery (including a delay of gratification test) showed stronger reactions to unequal treatment. This suggests that an individual’s ability to forego a reward and/or to refuse performing an action requested by one’s cooperative partner (e.g. handing back a token or giving the paw to an experimenter) may affect its response to inequity. Parrots have been shown to wait for better rewards for up to 15 minutes^36–38^, albeit they apparently struggle to inhibit motor responses in certain other task^39^. Nonetheless, the same species investigated here have been shown capable of economic decision-making, which involved refraining from taking immediately available rewards when waiting was more profitable^40^. Thus, parrots should be equipped with the underlying cognitive abilities to pay attention to reward quality and control their actions, at least in non-social setups.

In line with results from other taxa, the parrots did not respond to differential effort. Our findings indicate that, although some subjects may be sensitive to inequity, it is the difference in outcome rather than effort that influences their decision-making. Interestingly, while chimpanzees^5,41^ and long-tailed macaques^42^, responded to inequity in terms of reward quality but not effort, crows and ravens were amongst the few species that exhibited inequity aversion in both contexts^23^. One of the reasons for the lack of reaction to differential effort may be that the effort in the present study, i.e. exchanging a token twice, may not have been perceived as sufficiently costly for the subjects. However, van Wolkenten and colleagues^43^ found that the inequity response of capuchin monkeys was more pronounced if subjects had to make only a small effort (one exchange) compared to a large effort (three exchanges) when exchanging for less preferred food. They therefore proposed that increasing the effort may outweigh reward inequity, resulting in a decreased inequity response^43^.

So far, only two studies have investigated inequity aversion in birds using the same token-exchange paradigm, thus allowing a direct comparison of results. Crows and ravens stopped exchanging tokens and accepting rewards if they received a reward of lower quality compared to their partner^23^. The underlying motivation for this refusal, however, remains obscure, since the study did not control for frustration effects, e.g. by implementing a condition similar to our food control, in which a high-quality reward is moved towards the subject’s neighbour compartment, but in the absence of a partner. Consequently, it cannot be excluded that, alternatively, the corvids’ behaviour might be explained by frustration of not receiving a visible high-quality reward, rather than frustration resulting from comparing each other’s respective outcomes. This alternative explanation is however unlikely, considering that the corvids also ceased to participate if the partners were rewarded equally but for a lesser effort (i.e. the subject exchanged once, the partner got the reward for free). More recently, an alternative explanation has been suggested to interpret the negative reactions of chimpanzees in an inequity task, namely the aforementioned ‘Social Disappointment Hypothesis’, according to which subjects’ food refusal expresses social disappointment in the experimenter rather than inequity aversion^13^. Whether the corvids (and other animals) respond towards the experimenter more than towards the unequal reward distribution per se can be explained by the ‘Social Disappointment Hypothesis’ remains unclear and needs further testing.

The other study that has assessed inequity aversion in birds, focused on sub-adult kea, thus a very distantly related parrot species to the species investigated here (belonging to one of the two other superfamilies than that of our species), and revealed that they were not sensitive to unequal treatment^21^. This concurs with our findings and raises the possibility that – in contrast to some corvids - inequity aversion may not have evolved in parrots. However, it is important to keep in mind that our conclusion (like those of most previous studies) bases upon a single test and that we cannot exclude that our lack of evidence for inequity aversion in parrots may be a result of the probably quite artificial test setting. In such test setting individuals do not interact with conspecifics but a human experimenter, and in which none of the birds could influence the food distribution. Future studies incorporating a more naturalistic and/or ecologically valid test setting in which different partners vary in their cooperativeness, ideally in a naturally occurring cooperative behavior, might reveal inequity aversion in parrots. Also to be verified by future studies may be to examine to what extent the new “joint goal procedure” which we implemented in order to render the social comparison (i.e. paying attention to the outcome of one’s cooperation partner) more salient, would have affected the findings in the previously tested species. In contrast to previous studies, in which subjects alternatingly exchanged the token with the experimenter and received a reward, we essentially forced both individuals into a cooperative situation, where they would only be rewarded once both subjects had returned their tokens. Yet, while we would have expected this methodological change to increase the likelihood of detecting inequity aversion, we found no evidence for it in the tested parrots. The finding that kea, which were tested without such a procedure showed no inequity aversion either, suggests that the procedure did not affect their reaction.

More systematic comparisons, also including the remaining superfamily Cacatuidae, are required to confirm this assertion and to examine to what extent inequity aversion may be affected by species differences in social structure, social dynamics and mating system. It has recently been pointed out that reacting negatively to unequal treatment may only be adaptive if finding ‘better’ cooperative partners outweighs the costs of staying in an unequal partnership^14,44^. According to this hypothesis, inequity aversion might only be found in species that can flexibly switch to new cooperative partners, without incurring bigger costs, hence not in long-term monogamous species^9^. For species living in long-term monogamous pairs, in which the partner represents the main, if not the only cooperation partner who strongly affects individual fitness, members of a pair exhibit a high mutual dependence, and switching partners may be very costly. Consequently, moderate inequity between the members of a pair should be tolerated while only extreme and/or long-term inequity could potentially lead to the termination of the partnership. This hypothesis clearly holds the assumption that monogamous species do not cooperate with individuals other than their mate. Indeed, although gregariously living, many life-long monogamous species might simply reject cooperating with unrelated group mates other than their mated partner^14^. However, this does not hold true for all long-term monogamous species, e.g. cooperative breeders, and it is likely that there is considerable variation^9^. In line with this argumentation, the adaptive value of inequity aversion in long-term monogamous species should vary depending on the degree to which these species cooperate additionally with group members other than their mate. The seeming difference (based on the few currently available data) between corvids and parrots, which are both characterised by long-term monogamy, requires further investigation and can only be speculated upon at this stage. It may be that the investigated corvid species cooperate not just with their long-term mate but also with other individuals in their group (at least throughout certain periods in their ontogeny), while cooperative interactions with other group members do not (or to a lesser extent) occur in the parrots studied so far. Thus, future studies should test this assumption both within and across long-term monogamous species and examine whether the importance of inequity aversion as a regulatory mechanism depends on the degree of cooperation with group-members other than the mate.

In sum, our results provide further data that parrots may not be inequity averse. They support the hypothesis that inequity aversion is only adaptive in species, which cooperate with multiple partners and can switch cooperative partners without bigger costs. Parrots show a high level of interdependence of their mated partner and thus cannot change partners in case of moderate inequity. Additional studies are required in order to find out whether adult parrots exhibit inequity aversion when tested with a non-mated group member and whether inequity aversion varies according to mating system and the extent to which individuals cooperate with group-members other than their long-term mate. Even though pair-bonding is the most prevalent mating system in parrots, some species do exhibit other mating systems (see^45^ for a review). Accordingly, parrot species, which exhibit other i.e. non-monogamous mating systems, such as the polygynous Eclectus parrots (*Eclectus* sp.) or Vasa parrots (*Coracopsis vasa*), as well as species that are cooperative breeders, such as the golden parakeet (*Guaruba guarouba*)^46^ or monk parakeet (*Myopsitta monachus*)^47^, ought to be tested, in order to understand whether inequity aversion did not evolve in the *Psittaciformes* order at all or whether it is linked to the social organization and mating system of species.

## Materials and Methods

### Subjects

We tested 28 parrots of four different species (see Supplementary Table S1 for individual details): Eight great green macaws (5 F/1 M), six blue-throated macaws (all males), six blue-headed macaws (4 F; 2 M), and eight African grey parrots (6 F/2 M) ranging between 2 and 5 years of age. All parrots were hand-raised and subsequently socialized in groups in the Loro Parque Fundacíon, Tenerife, Spain.

#### Housing conditions

All parrots were group-housed in semi-outdoor aviaries at the Max-Planck Comparative Cognition Research Station in the Loro Parque in Puerto de la Cruz, Tenerife. Details on the aviary sizes for the study species and housing conditions as well as general set-up provided here have been described previously (see refs.^39,40^). The blue-throated macaws and the great green macaws were housed in eight aviaries, divided by species and age into five groups of two to eight individuals. Six of these aviaries were 1.8 × 7 × 3.6 m (width × length × height), and the remaining aviaries were 2 × 7 × 3.6 m and 1.5 × 7 × 3.6 m, respectively. These aviaries were interconnected by 1 m × 1 m windows, which could be closed when desired. The blue-headed macaws were housed together in a separate indoor area (28.61 m^2^) with access to a smaller outdoor area and the African grey parrots were housed together in another separate outdoor aviary (21.41 m^2^). All aviaries had at least one side open to the outside, so they followed a natural light schedule and were also kept to ambient outdoor temperature, but they were additionally lit with Arcadia Zoo Bars (Arcadia 54 W Freshwater Pro and Arcadia 54 W D3 Reptile lamp) to ensure sufficient exposure to UV light. All aviaries were situated within the same building as the testing chambers (described below).

The parrots were fed twice a day with a mix of fruits and vegetables shared between all the birds and in the evening, after test sessions, they received an individual amount of seeds calculated considering the amount of rewards (walnuts and seeds) they had received during the tests. Water was available *ad libitum*.

All the subjects had participated in several different social and physical cognition tests previously, so they were fully accustomed to experimental procedures, i.e. to the testing rooms, to the transport cages used for moving the birds between the aviary and the testing area, and to be handled by people.

#### Setup

Training and testing took place in an indoor chamber of 2.5 × 1.5 × 1.5 m (height x width x length) equipped with lamps covering the birds’ full range of visible light (Arcadia 39 W Freshwater Pro and Arcadia 39 W D3 Reptile lamp). A sound-buffered one-way glass system permitted zoo visitors to see inside the rooms but did not allow the birds to see out.

**Figure 2:**
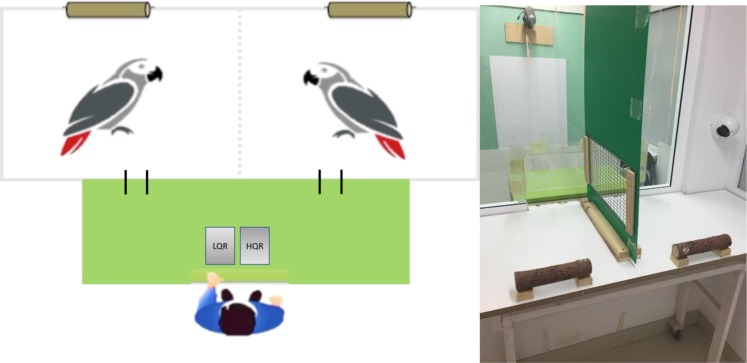
Schematic overview (left) of the experimental setup with the two birds in adjacent compartments (not to scale) and the experimenter opposite and a photograph (right) taken from the backside into the subjects’ compartments (right). The dividing wall between the birds and the experimenter was made of transparent Plexiglass and contained two exchange holes so that both birds could exchange tokens against food with the experimenter who faced them sitting at a green table opposite onto which two transparent boxes containing the high and low quality reward were placed. The non-transparent plastic dividing wall between the birds had a wire-mesh-covered window, allowing visual, acoustic and very limited tactile contact, but prevented the birds from direct interaction. A white table served as floor in each compartment, at the height of the experimenter’s experimental table, and had a perch located at its back. The remaining test room behind the table was shut off by a curtain to prevent the birds from accessing their neighbour compartment (not shown in the photograph).

The test chamber consisted of one table (87 × 49 × 150 cm) and a perch system at the back (see Fig. 2). For all the sessions, the birds’ test chamber was divided into two equally sized (150 × 49 × 75 cm) compartments by a separation wall. The separation wall consisted of an opaque plastic part dividing the table with an opening in the lower part covered by a mesh and allowing the birds visual and acoustic but no tactile contact, and a curtain dividing the rest of the room. The front of the test chamber consisted of a Plexiglas panel with two circular holes, i.e. one per compartment (10 cm in diameter for the great green macaws, 7.5 cm in diameter for the blue-throated macaws and the African grey parrots, and 5 cm for the blue-headed macaws) (see Fig. 2). The subject could put its head though this ‘exchange hole’, in order to hand in the token to the experimenter. The exchange holes for the larger species were 14,5 cm and the ones for the smaller species were 15 cm apart from the middle separation wall, and in horizontal alignment. Metal washers (3.5 cm in diameter) were used as nonedible tokens. The exchange ‘token-food’ with the experimenter happened very closely to the middle separation wall so that the neighbour bird would witness it. Two separate transparent boxes half-filled with low (LQR) and high-quality rewards (HQR) respectively were always placed onto the back of the table, equally visible to both birds. The reward boxes were refilled after each session.

#### General procedure

The subjects were tested once or twice per day, in the morning between 9 am and 12 pm and afternoon between 2 pm and 5 pm from September 2016 to September 2017. To ensure sufficient motivation, tests only took place with a minimum of four hours after the last feeding or before feeding in case of the morning sessions. Each subject was paired with a conspecific partner with whom it maintained a strong affiliative bond based on observational data collected prior to the start of the experiment (see Supplementary Table S1 for the dyad composition). Wherever possible, the age and the sex of the partners were matched, but the affiliative relationship had priority. In the four social test conditions, the subject always exchanged tokens with the experimenter after the partner had exchanged it first, whereas in the two non-social control conditions (Food control, FC, and Effort control, EC, see Table 2a,b further below for description) the exchange between the experimenter and the subject occurred after an equivalent delay of 5 sec. Prior to testing, each bird was randomly assigned to one fixed compartment side for the whole duration of the experiment, hence irrespective of their current role (based on who exchanged first; see explanation below), to avoid any confusion for the subjects in addition to their role switching.

#### Food preference test

Before testing, all subjects participated in a food preference test to verify their preference for a piece of walnut (high quality reward; HQR) over a single white sunflower seed (or one-quarter of a pumpkin seed for one African grey parrot dyad) (low quality reward; LQR). The subject was offered the two reward types simultaneously by the experimenter in different hands (both hands kept together in front of the hole). Only one choice was allowed and as soon as the bird took one reward, the other hand was removed. Twelve-trial sessions were conducted, with the sides of the reward types (i.e. left, right) alternating. A reward was considered preferred if the subject chose it at least 83% of the time over the alternative (10 out of 12 choices, two-sided binomial test, p < 0.039). All subjects preferred the walnut over the seed and reached criterion in their first session. In order to ensure that the birds would work for the LQR, the birds were requested to exchange the token for pieces of LQR 12 times in a row before the food preference test.

#### Training and familiarization

All birds were trained to trade in tokens for food from a previous study^36^ through positive reinforcement. The general procedure required the birds to pick up a token provided by the experimenter (i.e. pushed into the bird’s compartment through a gap under the Plexiglas panel in the front) and return it to the experimenter’s open hand in exchange for a reward. The experimenter solicited the exchange by moving her open hand (palm facing upwards) in front of the hole in the Plexiglas panel and keeping it there. Once the birds had released the token through the hole into the palm, the experimenter closed the hand around the token, placed the hand in sight of the birds in front of the exchange hole and repeated the procedure with the second bird. Once the second bird returned the token, the experimenter withdrew both hands. The experimenter then took the rewards from the transparent food boxes in front of them and delivered the respective rewards to the birds simultaneously according to the procedure described in the following.

For the present study and in contrast to all previous token exchange studies, the subjects were trained to a “joint goal procedure” before they proceeded to testing, i.e. they learned that they would only get the rewards once both individuals tested in a dyad had returned their tokens, so that they actually had to “cooperate”. After the first bird (i.e. the partner) received the token, the experimenter waited for 3 s before requesting the token back. If the partner successfully returned the token, the second bird (subject) got its turn receiving and returning its token; only after the subject had returned the token too, the experimenter handed out the rewards to both of them simultaneously (for the detailed description of the training and the criterion they had to meet see Supplementary Information). For this purpose, the experimenter picked up a piece of reward (LQR or HQR depending on the condition) with the left hand to deliver the reward to the left compartment and concurrently with the right hand to the right compartment. Before giving the two rewards to the two birds, they were shown to them close to the middle compartment on the two outstretched hands joined together for 3 s before each hand was moved to an exchange hole simultaneously (see Supplementary Video). An exchange was defined as successful if the subject returned the token within 30 s and ate the offered reward within 15 s. A failure to exchange occurred if the subject either failed to return the token within the allotted 30 s or dropped the token from the table instead of placing it in the experimenter’s hand. If the subject refused to exchange the token, only the partner got the reward (i.e. the hand that would otherwise deliver the reward to the subject remained motionless on the experimenter’s lap). In this case the experimenter held their hand in the middle (more on the side of the bird that would be rewarded) and handed the reward out to the partner. If the partner (the first bird to exchange) refused to exchange the token, this trial was considered invalid and interrupted and was repeated at the end of the session. If the partner refused to exchange two times in a row or four times in total, the session ended and was repeated the next day. When such an exchange refusal occurred, the trial ended and next started after 10 sec. A reward refusal occurred when the subject returned the token but failed to eat the reward within the 15 s of it being offered. If a bird was not attentive after it was handed the token, the experimenter called the name of the bird after 10 s and 20 s to attract the bird’s attention. Once established, this “joint goal procedure” was used throughout the subsequent test sessions.

#### Test conditions

Each bird participated in six test conditions, once in the subject role and once as the partner (see Table 1 for description). The role was indicated by who started the trial. The partner always started followed by the subject, ensuring the birds knew their respective roles. The birds always stayed in the same testing compartment, however the roles (i.e. the exchange order) and the condition changed pseudorandomly and in a counterbalanced manner across subjects (i.e. mixing all the conditions and roles, see Table S2 for an example). A criterion for randomization was that no subject was tested in the same role in the same condition across consecutive sessions. Additionally, the unequal condition could not follow the equal high condition. This ensured that the birds did not respond frustratedly because they were expecting a higher reward based on previous higher rewards.

Each condition consisted of three sessions of 12 trials each resulting in 36 trials in total per condition. The three sessions of each condition were intermixed and the order of the condition, session and the role of the bird were pseudorandomized and counterbalanced (see Table S2 in the Supplementary Information). Per day up to two sessions could be performed.

For a summary of the predictions for the three main variables in the experimental conditions according to the hypothetical framework of inequity aversion see Table 2.

### Ethical standards

All applicable international, national, and/or institutional guidelines for the care and use of animals were followed. In accordance with the German Animal Welfare Act of 25th May 1998, Section V, Article 7 and the Spanish Animal Welfare Act 32/2007 of 7th November 2007, Preliminary Title, Article 3, the study was classified as non-animal experiment and did not require any approval from a relevant body.

## Analyses

All tests were recorded, and the videos were coded using a video scoring software Solomon Coder (© 2015 by András Péter). The following behaviours were coded for each test: number of exchanges (=token placed in experimenter’s hand) and refusals (=token not returned within 30 s), numbers of reward refusals (=reward not consumed within 15 s), latency to exchange token (=time from requesting the token until it was placed in the experimenter’s hand), and latency to accept rewards (=time from offering the reward to the bird until it took it out of the experimenter’s hand). An additional fourth coder coded 10% of the videos from each species, to assess reliability between coders (*Ara ambiguus*: Cohen’s kappa: *k* = 1.0, ICC (consistency): all > 0.95; *Ara glaucogularis*: Cohen’s kappa: *k* = 1.0, ICC (consistency): all = 1.00; *Primolius couloni*: Cohen’s kappa: *k* = 1.0, ICC (consistency): all > 0.99; *Psittacus erithacus*: Cohen’s kappa: *k* = 1.0, ICC (consistency): all > 0.87).

Analyses were conducted in R^48^ utilizing the packages ‘lme4’^49^ and ‘nlme’^50^ for linear mixed models. Two-sided binomial tests were used to analyse food preferences against chance level (50%). We ran generalized linear mixed models (GLMMs) with a binomial distribution and logit-error function. The number of exchanges and refusals was used as response variable, while test condition and session were set as fixed effects. Identity and dyad were included as random effects. Wald χ^2^ tests were used to detect effects in the GLMMs. Moreover, linear models (LMs) with either log-transformed or 1/square-root transformed response variables and generalized least square models (GLS) (in case variable could not be normalized) were run using the latency to exchange and the latency to accept the reward as response variables respectively including test condition and session number as fixed effects. In this case, Anovas were used to detect effects in the LMs and GLSs. All model assumptions (i.e. normality, sphericity, overdispersion) were checked prior to running the model. Models were selected based on Akaike Information Criteria to find the best fit.

## Supplementary information

Supplementary Information

IA_Experimental conditions

## Data Availability

All data generated or analyzed during this study are included in this published article (and its Supplementary Information files).
