# Supplementary material for: Parrots do not show inequity aversion
Source: Sci Rep. 2019 Nov 11;9:16416. doi: 10.1038/s41598-019-52780-8 (PMC6848082; doi:10.1038/s41598-019-52780-8)
Supplement: Supplementary file 1 — Supplementary Information [file 41598_2019_52780_MOESM1_ESM.pdf]

Parrots do not show inequity aversion

Anastasia Krasheninnikova[1,2], Désirée Brucks[1,2], Nina Buffenoir[2], Dániel Rivas Blanco[2], Delphine Soulet[2], Auguste von Bayern[1,2,3]

[1] Max-Planck-Institute for Ornithology, Eberhard-Gwinner-Str., 82319 Seewiesen, Germany

[2] Max-Planck Comparative Cognition Research Station, Loro Parque Fundacíon, 38400 Puerto de la Cruz, Tenerife, Spain

[3] Department of Biology, Ludwig-Maximilians-University of Munich, 82152 Planegg-Martinsried, Germany

Supplementary Information

Training: Overview of the token exchange training steps.

Training token exchange:

All birds were also familiar with the token exchange procedure from a previous study (Krasheninnikova et al. unpublished data). For this study, token exchange had been trained via gradual shaping. The parrots were rewarded for behaviours that were incrementally closer to the desired exchange behaviour, which consisted of picking up the token and putting it in a metal box in the experimenter's hand through a hole in the Perspex panel between the two chambers. If the parrot returned the token correctly it was rewarded with a piece of walnut (approx. 0.9 g). The training finished after 15 correct exchanges within 15 trials had been achieved.

Training procedures:

All birds were trained in four training steps once they had completed the food preference tests.

Step 1: The goal of the first training step was to get the birds used to exchanging a token for food multiple times in a row. The birds were positioned in the test room in pairs separated in the two chambers and were alternately asked to exchange the token for food (i.e. both birds exchanged 16 times). We used two types of reward: high- (a piece of walnut; HQR) and a low-quality reward (a sunflower seed; LQR). Two types of sessions (each session consisting of 16 trials) were conducted, one in which the birds received HQR for the token and another session in which the birds received LQR. Session type varied pseudo-randomly (no more than two sessions of the same type in a row; 1-2 sessions a day). Individual birds stayed in the same compartment throughout all test sessions, but the order in which they exchanged with the experimenter alternated across sessions. For example, in session one bird A is asked to exchange the token first, then bird B, while in session two bird B is asked first followed by bird A. The birds were rewarded with the respective reward directly following the successful exchange. We conducted five training sessions for each food type (HQR, LQR), thus 10 training sessions.

Step 2: In the second training step, the “joint goal procedure”, both birds were requested to exchange their tokens before the rewards were handed out to both. Thus, the birds learned that they actually had to “work together” in order to receive rewards. After the first bird had successfully exchanged the

token, the second bird received its token and was asked to return it. Only then did the experimenter hand out the rewards to both birds simultaneously. The experimenter kept their hands together for two seconds in the middle, before distributing the reward to the birds, so that the birds could clearly see what reward they and their partner were about to receive. We conducted 12 sessions, 6 sessions for each type of reward (i.e. 6 session LQR, 6 session HQR for both birds).

Step 3: In the third training step, we used the same procedure as in second step, but now the birds received unequal rewards. This training step was included in order to exclude the possibility that the birds' reaction towards the inequity condition in the actual test was due to general novelty of this situation (i.e. that the partner suddenly gets another type of reward). Importantly, there was a key difference to the test: In this step the birds both got an equal amount of HQR and LQR per session, i.e. HQR in 8 trials and LQR in 8 trials. In each trial, one of the birds got the HQR and the other the LQR for exchanging a token. The order of trials was counterbalanced and pseudorandomised, with the rule that the birds could not get the same reward more than twice in a row. Here both rewards were always visible for the birds (two identical transparent plastic boxes were filled with rewards and placed close to each other in the middle of the table, on the table edge closer to the experimenter). Two such sessions of 16 trials were conducted before proceeding with the final training step.

Step 4: In the last training step the birds learned that they had to wait for three seconds before they were allowed to exchange the token (i.e. the experimenter placed the token into the chamber, removed the hand, and waited three seconds before requesting the bird to exchange). The birds had to successfully exchange tokens (= place token inside of experimenter's hand) in 10 out of 12 trials in two consecutive sessions before proceeding with the test. If a bird failed to exchange correctly, the trial ended and the next trial begun 10 seconds later. In this step, the birds were trained individually, in the test room without a separation wall. All individuals met the criterion within 5 sessions.

Table S1. Dyad composition, sex, year of birth and relatedness of parrots participating in the study (N = 28).

Species	Dyad	Name	Sex	Year of birth	Siblings
A. ambiguus	1	Alba	F	2014	yes
		Enya	F	2014	yes
	2	Luna	F	2014	yes
		Hagrid	M	2014	yes
	3	Madame	F	2014	no
		Rita	F	2014	no
	4	Acorn	F	2013	no
		Hazel	F	2011	no
A. glaucogularis	1	Baloo	M	2014	yes
		Paco	M	2014	yes
	2	Long John	M	2013	no
		Mowgli	M	2014	no
	3	Mr. Huang	M	2013	yes
		Charlie	M	2014	yes
P. couloni	1	Andromeda	F	2014	no
		Neptune	F	2014	no
	2	Callisto	F	2014	yes
		Lupita	F	2014	yes
	3	Mars	M	2014	yes
		Mercury	M	2014	yes
P. erithacus	1	Bella	F	2014	no
		Sensei	M	2014	no
	2	Jack	M	2014	yes
		Nikki	F	2014	yes
	3	Jelo	F	2014	no
		Kimmi	F	2014	no
	4	Lizzy	F	2014	yes
		Nina	F	2014	yes

Table S2. Example for pseudo-randomisation of the conditions and roles for one dyad (Jack-Nikki)

Total Sessions	Condition	Repetition	Subject	Partner
1	Unequal	1	Jack (L)	Nikki (H)
2	Equal Low	1	Nikki (L)	Jack (L)
3	Unequal Effort	1	Nikki (L)+effort	Jack (L)
4	Unequal	2	Jack (L)	Nikki (H)
5	Equal High	1	Nikki (H)	Jack (H)
6	Unequal Effort	2	Nikki (L)+effort	Jack (L)
7	Effort Control	1	Jack (L)+effort	---
8	Equal Low	1	Jack (L)	Nikki (L)
9	Effort Control	1	Nikki (L)+effort	---
10	Equal High	1	Jack (H)	Nikki (H)
11	Equal Low	2	Nikki (L)	Jack (L)
12	Effort Control	2	Jack (L)+effort	---
13	Equal Low	2	Jack (L)	Nikki (L)
14	Unequal	1	Nikki (L)	Jack (H)
15	Equal High	2	Jack (H)	Nikki (H)
16	Unequal Effort	3	Nikki (L)+effort	Jack (L)
17	Food Control	1	Jack (L)	--- (H)
18	Food Control	1	Nikki (L)	--- (H)
19	Equal Low	3	Jack (L)	Nikki (L)
20	Equal High	2	Nikki (H)	Jack (H)
21	Unequal Effort	1	Jack (L)+effort	Nikki (L)
22	Effort Control	3	Jack (L)+effort	---
23	Equal Low	3	Nikki (L)	Jack (L)
24	Unequal	2	Nikki (L)	Jack (H)
25	Equal High	3	Jack (H)	Nikki (H)
26	Unequal Effort	2	Jack (L)+effort	Nikki (L)
27	Food Control	2	Nikki (L)	--- (H)
28	Effort Control	2	Nikki (L)+effort	---
29	Unequal	3	Jack (L)	Nikki (H)
30	Equal High	3	Nikki (H)	Jack (H)
31	Unequal Effort	3	Jack (L)+effort	Nikki (L)
32	Food Control	3	Nikki (L)	--- (H)
33	Food Control	2	Jack (L)	--- (H)
34	Effort Control	3	Nikki (L)+effort	---
35	Food Control	3	Jack (L)	--- (H)
36	Unequal	3	Nikki (L)	Jack (H)

L = low quality reward; H = high quality reward; effort = required to exchange token twice; --- = no partner present
